# Does the transfer of a poor quality embryo with a good quality embryo benefit poor prognosis patients?

**DOI:** 10.1186/s12958-020-00656-2

**Published:** 2020-09-30

**Authors:** Wenjie Wang, Jiali Cai, Lanlan Liu, Yingpei Xu, Zhenfang Liu, Jinghua Chen, Xiaoming Jiang, Xiaohua Sun, Jianzhi Ren

**Affiliations:** Reproductive Medicine Center, Xiamen University Affiliated Chenggong Hospital, Wenyuan Road No.94, Siming District, Xiamen, Fujian 361001 People’s Republic of China

**Keywords:** Poor quality embryo, Good quality embryo, Single embryo transfer, Poor prognosis patients, Propensity score matching

## Abstract

**Background:**

While single embryo transfer (SET) is widely advocated, double embryo transfer (DET) remains preferable in clinical practice to improve IVF success rate, especially in poor prognosis patients with only poor quality embryos (PQEs) available in addition to one or no good quality embryos (GQEs). Furthermore, previous studies suggest PQE might adversely affect the implantation of a GQE when transferred together. This study aims to evaluate the effect of transferring an additional PQE with a GQE on the outcomes in poor prognosis patients.

**Methods:**

A total of 5037 frozen-thawed blastocyst transfer (FBT) cycles between January 2012 and May 2019 were included. Propensity score matching was applied to control for potential confounders, and we used generalized estimating equations (GEE) models to identify the association between the effect of an additional PQE and the outcomes.

**Results:**

Overall, transferring a PQE with GQE (Group GP) achieved significantly higher pregnancy rate (PR), live birth rate (LBR) and multiple pregnancy rate (MPR) than GQE only (group G). The addition of a PQE increased LBR in patients aged 35 and over and in patients who received over 3 cycles of embryo transfer (ET) (48.1% vs 27.2%, OR:2.56, 95% CI: 1.3–5.03 and 46.6% vs 35.4%, OR:1.6, 95% CI: 1.09–2.35), but not in women under 35 and in women who received less than 3 cycles of ET (48.7% vs 43.9%, OR:1.22, 95% CI: 0.93–1.59 and 48.3% vs 41.4%, OR:1.33, 95% CI: 0.96–1.85). Group GP resulted in significantly higher MPR than group G irrespective of age and the number of previous IVF cycles.

**Conclusions:**

An additional PQE does not negatively affect the implantation potential of the co-transferred GQE. Nevertheless, the addition of a PQE contributes to both live birth and multiple birth in poor prognosis patients. Physicians should still balance the benefits and risks of DET.

## Introduction

Multiple pregnancies are considered as the most serious adverse outcome related to ART, associated with increased risks of maternal and fetal morbidity [1]. The most recommended way to minimize the incidence of multiple pregnancies and the associated risks is single embryo transfer [2]. While a global increasing trend of the use of single blastocyst transfer (SBT) has been reported, recent large scale data also suggested that transferring more than one embryo remained relatively common in clinical practice [3]. For instance, 15,741 of more than 54,000 IVF cycles performed in UK were transferred with more than one embryos, resulting a multi birth rate over 30% [3].

Transferring more than one embryos is usually considered in patients with unfavorable prognosis, such as advanced age or multiple failed previous cycles [4]. Such patients, however, may only have limited choice of embryos available for transfer. While only poor quality embryos (PQEs) available in addition to one or no good quality embryos (GQEs), physicians may confront a dilemma in clinical practice: whether one should transfer an additional PQE with a GQE to maximize the treatment success.

On the other side, growing evidence suggest that the communication between the embryo and the endometrium occurs during implantation [5, 6]. The endometrium which is characterized as sensor of embryo quality might be able to distinguish signals from competent embryo and developmentally abnormal embryo, and convert the signals into a go or no-go endometrial response. It is likely that the PQE might send aberrant and harmful signals to the endometrium, resulting in a rejection response as well as detrimental reproductive outcomes of the co-transferred GQE.

According to a recent reported study, Dobson et al. suggested that transferring a poor-quality embryo along with a top-quality embryo increases the multiple birth rate without increasing the live birth rate [7]. However, previous studies reported conflicting results when comparing pregnancy rate (PR) and LBR of SBT with double blastocyst transfer (DBT) in frozen-thawed cycles. Some investigators demonstrated no significant differences in PR and LBR between SBT and DBT [8, 9], while some studies indicated SBT met a lower PR and a lower LBR compared to DBT [10, 11]. Several confounding factors that may affect the live birth rate, such as total number of blastocysts available, ovarian reserve of the patients, ovarian stimulation protocols, insemination method and cryopreservation may contribute to the inconsistency. Furthermore, few study have stratified the patients with poor prognosis from those without.

The objective of this study was to evaluate the effect of DBT with one GQE plus one PQE on the outcomes in patients undergoing frozen-thawed cycles in a propensity score matching design. Additionally, patients were stratified according to advanced age and repeated failure to investigate a potential modification of poor prognosis.

## Materials and methods

### Study design and patients

This was a retrospective study performed at Xiamen University Affiliated Chenggong Hospital. Patients received either DBT with a PQE and a GQE or SBT with only a GQE during frozen-thawed blastocyst transfer (FBT) cycles in the period between January 2012 and May 2019 were included. Exclusion criteria were: (a) Blastulation on day 7 (b) Blastocysts derived from vitrified oocytes or vitrified cleavages. Cycles with missing data and women lost to follow-up were secondarily excluded. Patients undergoing SBT with a GQE were defined as group G and DBT with a GQE plus a PQE were defined as group GP. Some patients contributed multiple cycles in this study. Institutional Review Board approval was obtained from the Ethics Committee of the Medical College of Xiamen University.

### Treatment protocol and embryo quality assessment

In stimulation cycles, all patients were treated with agonist or antagonist protocol with the use of FSH or hMG as previously described [12]. The initial and ongoing dosage was adjusted according to the patient’s age, antral follicle count (AFC), BMI, and ovarian response. Ovarian response was monitored by means of transvaginal ultrasound measurements of follicular growth and serum E2 level every 1–3 days. When at least one follicle reached a mean diameter of 18 mm as determined by ultrasound, hCG was administrated. Oocyte retrieval was scheduled for 34–36 h after hCG injection and was carried out under transvaginal ultrasound guidance.

Conventional IVF or ICSI was carried out depending on semen parameters and previous fertilization histories. In IVF cycles, cumulus-oocyte complexes were inseminated with approximate 1.5–3 X 10^5^ progressively motile spermatozoa in fertilization culture medium (K-SIFM, Cook) for 4 h. Oocytes for ICSI were denuded 2 h after ovum pickup, and sperm microinjection was performed 4 h after retrieval. Fertilization was checked about 17 h post insemination/injection and was determined by the presence of two pronuclei (2PN).

All embryos were cultured under mineral oil in traditional incubators (C200, Labotect) at 37 °C, 6% CO2, 5% O2. Cook IVF media (Cook Medical) was used for cleavage-stage embryos (K-SICM) and blastocysts (K-SIBM) culture in the form of microdrop of 20 μl. On day 3, evaluation of embryo quality included the number of blastomere, the degrees of fragmentation and the uniformity of blastomeres. Cleavages were determined for fresh embryo transfer or blastocyst culture, and then were placed in blastocyst culture medium(K-SIBM). The quality of blastocysts were evaluated on Day 5 or Day 6 based on the Gardner and Schoolcraft grading system, and the score was dependent on blastocyst expansion, inner cell mass (ICM) development and trophectoderm (TE) appearance [13]. Good quality embryos were blastocysts graded as AA, AB, BA and BB with expansion grade ≥ 3, while poor quality embryos were those defined as AC, CA, BC, CB and CC with expansion grade ≥ 3. In addition, Top quality embryo (TQE) were blastocysts graded as AA, AB and BA with expansion grade ≥ 4. Blastocysts were determined to be transferred on day 5 or vitrified for subsequent transfer. Blastocysts with poor morphological score (≤4CC) or low expansion grade (grades 1–2) were not considered for vitrification or transfer.

### Vitrification and thawing

For vitrification, the cryotop method was carried out as described by Kuwayama [14]. Briefly, blastocysts were equilibrated for 3–5 min in equilibration solution (ES:7.5% dimethyl sulfoxide and 7.5% ethylene glycol), and were then placed into in vitrification solution (VS:15% dimethyl sulfoxide, 15% ethylene glycol, 10 mg/mL Ficoll-70, and 0.6 M sucrose). After 30–40 s in VS, embryos were transferred on the cryotop strip and plunged into liquid nitrogen immediately. For thawing, blastocysts were directly immersed into thawing solution (TS) containing 1 M sucrose at 37 °C for 1 min, then was sequentially incubated in each of the following solutions for 3 min: 0.5 M sucrose, 0.25 M sucrose and sucrose-free TS. Then the embryos were placed in blastocyst culture medium (K-SIBM, Cook) and cultured in an incubator at 37 °C with 6% CO2 until transfer. Survival of thawed embryos were assessed under an inverted microscope depending on whether blastocysts showed a severely damaged cellular content or not.

### Endometrial preparation and embryo transfer

Three main types of endometrial preparation protocols were applied: the natural cycle (NC), hormone replacement treatment (HRT) cycle with or without GnRH downregulation. In NC cycles, growth of follicles was monitored under transvaginal ultrasonography from cycle day 9 to 11. LH and estradiol were measured every 3 days after the diameter of leading follicle ≥14 mm. When a spontaneous LH surge was detected and ovulation occurred, intramuscular progesterone injections (40 mg/day) were started at the day of ovulation (set as day 0) and FBT was scheduled on the 5th day after ovulation.

Or when a spontaneous LH surge was detected but ovulation was not monitored to occur, the following second day was considered as ovulation day (day 0) and FBT was scheduled on the 5th day after ovulation. HRT was performed with 6 mg oral estradiol valerate daily from cycle day 1 to day 14. Progesterone injection (40 mg) was administrated (set as day 0) as soon as the endometrial thickness reached 7–8 mm and then FBT was scheduled after 6 days of progesterone therapy. As for HRT with GnRH- downregulation cycles, GnRH agonist was initiated on day 1 of the menstrual cycle. And on day 1 of subsequent menstruation, estrogen stimulation was started as HRT cycles without GnRH agonist. Embryo transfer was performed with a Guardia Access Embryo Transfer catheter (K-JETS-7019-SIVF, Cook, IN, USA) under transabdominal ultrasound guidance. Luteal support continued until 10 weeks of pregnancy.

### Statistical analysis

The primary outcomes were live birth rate (LBR) and multiple pregnancy rate (MPR). Secondary outcomes of the study included clinical pregnancy rate (PR) and miscarriage rate (MR).

The baseline characteristics were compared between the two groups. The normality of continuous variables was examined by normality plots and Shapiro-Wilk test. Since none of the Continuous variables studied demonstrated normal distribution by both tests, they are presented as medians (first quartile, third quartile), while categorical variables are presented as n (%). Continuous variables were analyzed by Mann-Whitney U test, and categorical variables were analyzed using Chi-square test or Fisher’s exact test. All values were two tailed and *P* < 0.05 was considered to be significant. All analyses were performed by using SPSS (version 22, IBM).

As two groups were not randomly assigned in clinical practice, potential confounders and selection biases were accounted for by propensity score matching [15]. Propensity scores were calculated using logistic regression based on potential variables related to the outcome [16]. The variables included maternal age, paternal age, maternal BMI, parity, gravidity, duration of infertility, cause of infertility, baseline FSH, antral follicle count (AFC), ovarian stimulation protocol, insemination methods, endometrial preparation protocol, endometrial thickness, number of blastocyst vitrified, cycles of ET, day of blastocyst transferred and the proportion of using top quality blastocysts. A one-to-one nearest neighbor matching method without replacement was performed to match data between group G and group GP with a caliper width equal to 0.03 [17]. In order to investigate the effect of group GP in women aged 35 and over and in women received at least 3 cycles of ET, two groups were stratified by age and ET order, and PS matching of each subgroup was performed separately. PS matching was performed by using MatchIt package in R software.

A generalized estimating equations (GEE) model was conducted to evaluate the association between the effect of an additional PQE and outcomes due to including patients contributing multiple cycles [11]. To further verify the results, multivariate GEE models were performed using pre-matching data to adjust for aforementioned confounders.

## Results

A total of 5037 women were included in this study. Group G consisted of 4484 patients and Group GP included 553 patients. After propensity score matching, 520 patients in group G were matched by their counterparts in group GP.

Patients’ overall demographics and baseline IVF characteristics were presented in Table 1 (left panel). Significant differences were observed in terms of paternal age, AFC, duration of infertility, No. of blastocysts vitrified, ovarian stimulation protocol, endometrial preparation protocol, cycles of ET, day of blastocyst transferred and the proportion of using top quality blastocysts between two groups (*P* < 0.05). Comparison after PS matching was also listed in Table 1 (right panel), all the baseline characteristics became very comparable between two groups (*P* > 0.05). The distributions of the standard differences before and after PS matching were plotted (Figure S1). Standard difference < 0.1 was used as the threshold to indicate a negligible difference in the prevalence of a covariate between exposure groups.

**Table 1:** Patient characteristics of group G and group GP before and after PS matching

Variable	Before matching			After matching		
	G (***n*** = 4484)	GP (***n*** = 553)	***P***	G(***n*** = 520)	GP (***n*** = 520)	***P***
Maternal age	30 [27–33]	30 [27–33]	0.327	30.5 [28–34]	30 [27–33]	0.235
Paternal age	32 [29–35]	32 [29–36]	**0.001**	32 [29–36]	32 [29–35]	0.991
Duration of infertility	3.2 [2–5]	4 [2–6]	**0.014**	3.75 [2–6]	4 [2–6]	0.805
BMI (kg/m^2^)	20.8 [19.2–22.4]	20.8 [19.31–22.5]	0.47	21 [19.23–22.6]	20.8 [19.3–22.6]	0.77
PCOS (%)	427 (9.5)	43 (7.8)	0.183	44 (8.5)	41 (7.9)	0.734
Endometriosis (%)	396 (8.8)	58 (10.5)	0.199	57 (11)	54 (10.4)	0.763
Tubal factor (%)	2952 (65.8)	348 (62.9)	0.175	317 (61)	329 (63.3)	0.443
Male factor (%)	827 (18.4)	108 (19.5)	0.535	110 (21.2)	101 (19.4)	0.488
Parity (%)						
0	3736 (83.3)	471 (85.2)	0.268	427 (82.1)	443 (85.2)	0.18
≥ 1	748 (16.7)	82 (14.8)		93 (17.9)	77 (14.8)	
Gravidity (%)						
0	2281 (50.9)	288 (52.1)	0.706	272 (52.3)	270 (51.9)	0.977
1	1185 (26.4)	137 (24.8)		126 (24.2)	129 (24.8)	
≥ 2	1018 (22.7)	128 (23.1)		122 (23.5)	121 (23.3)	
Baseline FSH	6.52 [5.61–7.6]	6.58 [5.77–7.77]	0.056	6.66 [5.69–7.68]	6.67 [5.78–7.82]	0.573
AFC	12 [8–16]	11 [8–15]	**0.005**	11 [8–15]	11 [8–15]	0.869
Ovarian stimulation (%)						
Non-Agonist	386 (8.6)	63 (11.4)	**0.03**	68 (13.1)	62 (11.9)	0.574
Agonist	4098 (91.4)	490 (88.6)		452 (86.9)	458 (88.1)	
Insemination (%)						
IVF	3264 (72.8)	386 (69.8)	0.137	352 (67.7)	362 (69.6)	0.504
ICSI	1220 (27.2)	167 (30.2)		168 (32.3)	158 (30.4)	
Endometrial thickness	8.5 [7.6–9.8]	8.7 [7.8–9.8]	**0.031**	8.7 [7.7–10]	8.6 [7.8–9.8]	0.961
No.of blastocyst vitrified	3 [1–5]	1 [0–2]	**< 0.001**	0 [0–2]	1 [0–2]	0.065
Cycles of ET (%)						
1	1872 (41.7)	66 (11.9)	**< 0.001**	60 (11.5)	66 (12.7)	0.488
2	1800 (40.1)	236 (42.7)		217 (41.7)	230 (44.2)	
≥ 3	812 (18.1)	251 (45.4)		243 (46.7)	224 (43.1)	
Endometrial Preparation (%)						
NC	2158 (48.1)	234 (42.3)	**< 0.001**	212 (40.8)	224 (43.1)	0.726
HRT	1644 (36.7)	142 (25.7)		148 (28.5)	139 (26.7)	
HRT with GnRHa	682 (15.2)	177 (32)		160 (30.8)	157 (30.2)	
Day of blastocyst (%)						
Day5	4008 (89.4)	271 (49)	**< 0.001**	281 (54)	270 (51.9)	0.494
Day6	476 (10.6)	282 (51)		239 (46)	250 (48.1)	
FBT with TQE (%)	2100 (46.8)	103 (18.6)	**< 0.001**	109 (21)	100 (19.2)	0.486

Data are presented as median [first quartile, third quartile] and n (%)

*PCOS* Polycystic ovary syndrome, *ET* Embryo transfer, *NC* Natural cycle, *HRT* Hormone replacement therapy, *FBT* Frozen blastocyst transfer, *TQE* Top quality embryo, blastocysts graded as AA, AB and BA with expansion grade ≥ 4

Table 2 shows the outcomes of both groups before and after PS matching. Group GP achieved significantly higher PR (57.3%vs47.3%, OR:1.51, 95% CI: 1.18–1.93), LBR (47.9% vs 41%, OR:1.33, 95% CI: 1.04–1.7) and MPR (30.5%vs2.4%, OR: 17.49, 95% CI: 7.49–40.81) than group G after PS matching. MR for group GP were similar to group G (15.4% vs 13.4%, OR: 1.18, 95% CI: 0.73–1.9).

**Table 2:** Overall outcomes of group G and group GP before and after PS matching

	Before matching			After matching		
	G (***n*** = 4484)	GP (***n*** = 553)	***P***	G(***n*** = 520)	GP (***n*** = 520)	***P***
Clinical Pregnancy	2607 (58.1)	313 (56.6)	0.489	246 (47.3)	298 (57.3)	**0.001**
OR (95% CI)	Ref	1.44 (1.18–1.76)	**< 0.001**	Ref	1.51 (1.18–1.93)	**0.001**
Multiple pregnancy	74 (2.8)	96 (30.7)	**< 0.001**	6 (2.4)	91 (30.5)	**< 0.001**
OR (95% CI)	Ref	18.56 (11.92–28.92)	**< 0.001**	Ref	17.49 (7.49–40.81)	**< 0.001**
Miscarriage	346 (13.3)	47 (15)	0.393	33 (13.4)	46 (15.4)	0.505
OR (95% CI)	Ref	0.94 (0.64–1.36)	0.729	Ref	1.18 (0.73–1.9)	0.513
Live birth	2232 (49.8)	263 (47.6)	0.325	213 (41)	249 (47.9)	**0.025**
OR (95% CI)	Ref	1.37 (1.11–1.68)	**0.003**	Ref	1.33 (1.04–1.7)	**0.024**

Data are presented as n (%). Comparisons were made using chi-square test or Fisher’s exact test as appropriate

ORs were adjusted for variables presented in Table 1 using multivariate GEE model before matching

ORs after matching were adjusted for propensity score

*OR* Odds ratio

The outcomes of both groups stratified by age using a cutoff of 35 years old were displayed in Table 3. After matching, in women less than 35 years of age, PR (58% vs 50.1%, OR:1.38, 95% CI: 1.05–1.82) and MPR (31.7% vs 1.9%, OR:23.81, 95% CI: 8.54–66.43) were significantly higher in group GP than in group G. However, there were no significant differences in MR and LBR between two groups (31.6% vs 27.4 and 48.7% vs 43.9%, respectively). Interestingly, as for women 35 years of age and over, not only PR and MPR, but also LBR (48.1%vs27.2%, OR:2.56, 95% CI: 1.3–5.03) were found significantly higher in group GP than in group G. Adjusted OR for multiple pregnancy in women aged 35 and over before PS matching was not given, because multivariate GEE model was not available when the incidence of multiple pregnancy is low in relation to 21 variables used in the adjustment model.

**Table 3:** Outcomes of group G and group GP stratified by 35 years of age before and after PS matching

	Before matching			After matching		
**Age < 35**	**G** (***n*** **= 3799**)	**GP (*n*** **= 452)**	***P***	**G(*n*** **= 419)**	**GP (*n*** **= 419)**	***P***
Clinical Pregnancy	2296 (60.4)	259 (57.3)	0.198	210 (50.1)	243 (58)	**0.022**
OR (95% CI)	Ref	1.35 (1.08–1.7)	**0.009**	Ref	1.38 (1.05–1.82)	**0.02**
Multiple pregnancy	69 (3)	84 (32.4)	**< 0.001**	4 (1.9)	77 (31.7)	**< 0.001**
OR (95% CI)	Ref	17.88 (11.36–28.15)	**< 0.001**	Ref	23.81 (8.54–66.43)	**< 0.001**
Miscarriage	267 (11.6)	38 (14.7)	0.152	23 (11)	36 (14.8)	0.223
OR (95% CI)	Ref	1.22 (0.81–1.84)	0.351	Ref	1.42 (0.81–2.48)	0.219
Live birth	2006 (52.8)	218 (48.2)	0.066	184 (43.9)	204 (48.7)	0.166
OR (95% CI)	Ref	1.21 (0.97–1.51)	0.098	Ref	1.22 (0.93–1.59)	0.159
**Age ≥ 35**	**G** (***n*** **= 685**)	**GP (*n*** **= 101)**	***P***	**G(*n*** **= 81)**	**GP (*n*** **= 81)**	***P***
Clinical Pregnancy	311 (45.4)	54 (53.5)	0.129	31 (38.3)	46 (56.8)	**0.018**
OR (95% CI)	Ref	1.93 (1.21–3.08)	**0.006**	Ref	2.17 (1.15–4.1)	**0.017**
Multiple pregnancy	5 (1.6)	12 (22.2)	**< 0.001**	1 (3.2)	12 (26.1)	**0.009**
OR (95% CI)	–	–	–	Ref	10.87 (1.4–84.62)	**0.023**
Miscarriage	79 (25.4)	9 (16.7)	0.166	9 (29)	7 (15.2)	0.143
OR (95% CI)	Ref	0.37 (0.16–0.88)	**0.024**	Ref	0.45 (0.15–1.37)	0.159
Live birth	226 (33)	45 (44.6)	**0.022**	22 (27.2)	39 (48.1)	**0.006**
OR (95% CI)	Ref	2.71 (1.63–4.5)	**< 0.001**	Ref	2.56 (1.3–5.03)	**0.006**

Data are presented as n (%). Comparisons were made using chi-square test or Fisher’s exact test as appropriate

ORs were adjusted for variables presented in Table 1 using multivariate GEE model before matching

ORs after matching were adjusted for propensity score

*OR* Odds ratio

Comparisons of two groups stratified by cycles of ET were listed in Table 4. For patients who received ET less than 3 cycles, group GP had no differences in PR, MR and LBR compared to group G. However, both PR (56.5% vs 42.2%, OR:1.79, 95% CI: 1.22–2.61) and LBR (46.6% vs 35.4%, OR:1.6, 95% CI: 1.09–2.35) were observed statistically higher in group GP when compared to group G in patients undergoing at least 3 times embryo transfer. MPR were consistently significantly higher in group GP than in group G (31.7%vs5.6%, OR:7.97, 95% CI: 3.6–17.63) regardless of ET cycles.

**Table 4:** Outcomes of group G and group GP stratified by 3 cycles of ET before and after PS matching

	Before matching			After matching		
ET<3	G (*n* = 3672)	GP (*n* = 302)	*P*	G(*n* = 290)	GP (*n* = 290)	*P*
Clinical Pregnancy	2217 (60.4)	171 (56.6)	0.2	143 (49.3)	164 (56.6)	0.081
OR (95% CI)	Ref	1.3 (0.99–1.7)	0.06	Ref	1.35 (0.97–1.89)	0.076
Multiple pregnancy	68 (3.1)	56 (32.7)	**< 0.001**	8 (5.6)	52 (31.7)	**< 0.001**
OR (95% CI)	Ref	17.66 (10.3–30.3)	**< 0.001**	Ref	7.97 (3.6–17.63)	**< 0.001**
Miscarriage	276 (12.4)	24 (14)	0.547	21 (14.7)	23 (14)	0.869
OR (95% CI)	Ref	0.94 (0.56–1.58)	0.823	Ref	0.97 (0.51–1.83)	0.915
Live birth	1919 (52.3)	146 (48.3)	0.19	120 (41.4)	140 (48.3)	0.095
OR (95% CI)	Ref	1.21 (0.92–1.58)	0.167	Ref	1.33 (0.96–1.85)	0.091
ET ≥ 3	G (*n* = 812)	GP (*n* = 251)	*P*	G(*n* = 223)	GP (*n* = 223)	*P*
Clinical Pregnancy	390 (48)	142 (56.6)	**0.018**	94 (42.2)	126 (56.5)	**0.002**
OR (95% CI)	Ref	1.7 (1.23–2.35)	**0.001**	Ref	1.79 (1.22–2.61)	**0.003**
Multiple pregnancy	6 (1.5)	40 (28.2)	**< 0.001**	2 (2.1)	34 (27)	**< 0.001**
OR (95% CI)	Ref	24.39 (8.99–66.16)	**< 0.001**	Ref	17.16 (4.05–72.78)	**< 0.001**
Miscarriage	70 (17.9)	23 (16.2)	0.638	14 (14.9)	20 (15.9)	0.842
OR (95% CI)	Ref	0.87 (0.49–1.55)	0.63	Ref	1.1 (0.52–2.33)	0.799
Live birth	313 (38.5)	117 (46.6)	**0.023**	79 (35.4)	104 (46.6)	**0.016**
OR (95% CI)	Ref	1.58 (1.14–2.2)	**0.006**	Ref	1.6 (1.09–2.35)	**0.017**

Data are presented as n (%). Comparisons were made using chi-square test or Fisher’s exact test as appropriate

ORs were adjusted for variables presented in Table 1 using multivariate GEE model before matching

ORs after matching were adjusted for propensity score

*OR* Odds ratio

A post-hoc power calculation demonstrated that the study sample size reached 61% power in overall groups, 78.9% power in advanced age subgroups and 67.3% power in repeatedly failed subgroups in the primary outcome after PS matching.

## Discussion

This study indicated that the transfer of an additional PQE along with a GQE did not have a detrimental effect on GQE. Conversely, DBT with one PQE with one GQE (group GP) achieved significantly higher PR, LBR, and MPR than SBT with only one GQE (group G). In patients younger than 35 years, the addition of a PQE significantly increased PR and MPR without increasing LBR, while the additional PQE increased not only PR and MPR but also LBR in patients aged 35 and over. Similarly, in patients who received <3 cycles of ET, only MPR were found significantly higher in the group GP, while in patients received ≥3 cycles of ET, significantly higher PR, MPR and LBR were observed in the group GP.

Emerging evidence suggest that embryo-endometrial crosstalk plays an imperative role in the implantation process [5, 6]. According to the novel concept, embryos might signal the endometrium with embryonic serine proteases, and the endometrium is intrinsically capable of mounting an implantation response that is tailored to individual embryos. It is likely that the response of the luminal epithelium transduces and amplifies signals from developmentally competent embryos, activating a gene network enriched in metabolic enzymes and implantation factors, through which renders the underlying decidual layer more receptive. Conversely, developmentally impaired embryos elicit an endoplasmic stress response in human decidual cells, resulting in selectively inhibiting the secretion of key implantation factors, such as interleukin (IL)-1b, − 6, − 10, − 17, and − 18, as well as eotaxin (CCL11) and HB-EGF. These observations imply that a poor-quality embryo may affect negatively endometrium receptivity and interfere the implantation of a simultaneously transferred good-quality embryo.

Several studies have evaluated whether the transfer of an additional PQE has an adverse impact on a GQE when transferred together. In one analysis, Wintner et al. [18] compared good-quality fresh embryo transfers versus good-quality embryo transfers together with a poor-quality embryo in a mix of day 3 and day5 transfers. They concluded a PQE did not negatively affect a GQE when transferred together and no statistically significant differences were found in LBR and MPR between two groups. Li et al. [19] compared the transfer of a PQE plus a GQE with the transfer of two GQEs. They found no differences in PR and LBR between two groups, which also indicated a PQE did not have an adverse influence on a GQE. Berkhout et al. [20] suggested the addition of a low-quality embryo in fresh Day 3 DET did not improve the ongoing pregnancy rate but increased multiple gestation rates in fresh DET. However, all these three studies concerned cleavage stage transfers, which might differ from FBT cycles [21].

In a recent study, Hill et al. [22] reported that the addition of a lower-quality blastocyst was not harmful to the implantation of a co-transferred good-quality blastocyst and resulted in increases in live births and multiple gestations. Their results were consistent to ours. Dobson and colleagues demonstrated DBT with one PQE plus one GQE did not increase LBR but increased MPR when compared with SBT with GQE only [7]. However, the only confounder they have adjusted for OR was age. There were likely some other factors which would confound for the results as other studies have reported [23, 24]. In contrast, El-Danasouri and colleagues found that transferring an impaired quality embryo along with a good quality embryo significantly lowered both the pregnancy rate and implantation rate, than transferring the good quality embryo alone [25]. However, their study did not demonstrate a statistically significant difference. Notably, aforementioned studies suggested that DBT increased a significantly higher MPR than SBT.

Two prior studies have compared the outcomes of DBT with SBT in advanced maternal age. One study found that DBT resulted in a higher live birth than SBT in patients aged 35 years and over in vitrified-warmed cycles [8]. Another study indicated elective SBT was associated with similar LBRs compared to the entire DBT cohort, but the subgroup of women who had elective DBT achieved a higher LBR in advanced maternal age [26]. Our study also found that DBT with one GQE plus one PQE achieved a higher LBR than SBT with only one GQE in women aged 35 and over but not in women under 35 years of age. Within DBT cohort, we noticed patients aged 35 and over and patients under 35 years old had similar PR, MPR, MR and LBR. However, in SBT cohort, women over 35 years old had a lower trend in PR and a statistically higher MR than women under 35 years old, leading to a significantly lower LBR. The increased miscarriage rate may possibly be associated with the prevalence of aneuploidy which rose along with maternal age [27]. The addition of a second PQE somehow seemed to offset the loss in DBT cohort with the advantage of one more shot. It indicated that an additional PQE did not negatively affect a GQE when transferred together and morphologically poor blastocysts still have the implantation potential.

One study has reported the comparison of DBT versus SBT in patients who have experienced repeated implantation failures (RIF). Ohgi et al. [28] suggested that DBT with one GQE plus one PQE did not increase PR when compared with SBT with only one GQE among patients with RIF. However, the small sample size of their study might be unlikely to detect differences between two groups. Besides, RIF is a complex problem and uterine, male, or embryo factors, or the specific type of IVF protocol can be related independent factors that confound for the results. Our data, to some extent, indicated that the addition of poor quality blastocyst did not adversely affect the endometrial receptivity as well as the implantation of the co-transferred GQE.

A main strength of our study is that it included the largest number of patients on this topic to-date. Second, PS matching was conducted to control for potential confounders which might have effects on the outcomes. It has been proofed that PS matching provides an approach to mimic random assignment as RCT and is superior to conventional regression-based methods in a real world observational study [15]. Third, the comparisons were not only performed in overall groups, but were also explored in advanced maternal age and in women received at least 3 cycles of ET.

Our study was limited by its retrospectively observational design, and patients’ information were previously recorded by hospital with some missing data. Though PS matching was performed to evaluate the effects of DBT with mixed quality embryo independently from other confounders, the sample decreased after PS and the loss of unmatched cases might have unforeseen effects. Accordingly, results by multivariable GEE models to adjust potential confounders before PS matching was presented relatively.

## Conclusion

Our study indicates that the transfer of an additional PQE does not negatively affect the implantation potential of the co-transferred GQE. Nevertheless, the addition of a PQE contributes to both live birth and multiple birth in poor prognosis patients. Physicians should still balance the benefits and risks of DET.

## Supplementary information

**Additional file 1: Figure S1.** The distributions of the standard differences before and after PS matching were plotted. Standard difference < 0.1 was used as the threshold to indicate a negligible difference in the prevalence of a covariate between exposure groups.

## Data Availability

Not applicable.
