# Supplementary material for: Does the transfer of a poor quality embryo with a good quality embryo benefit poor prognosis patients?
Source: Reprod Biol Endocrinol. 2020 Sep 30;18:97. doi: 10.1186/s12958-020-00656-2 (PMC7526391; doi:10.1186/s12958-020-00656-2)
Supplement: Supplementary file 1 — Additional file 1: Figure S1. The distributions of the standard differences before and after PS matching were plotted. Standard difference < 0.1 was used as the threshold to indicate a negligible difference in the prevalence of a covariate between exposure groups. [file 12958_2020_656_MOESM1_ESM.docx]

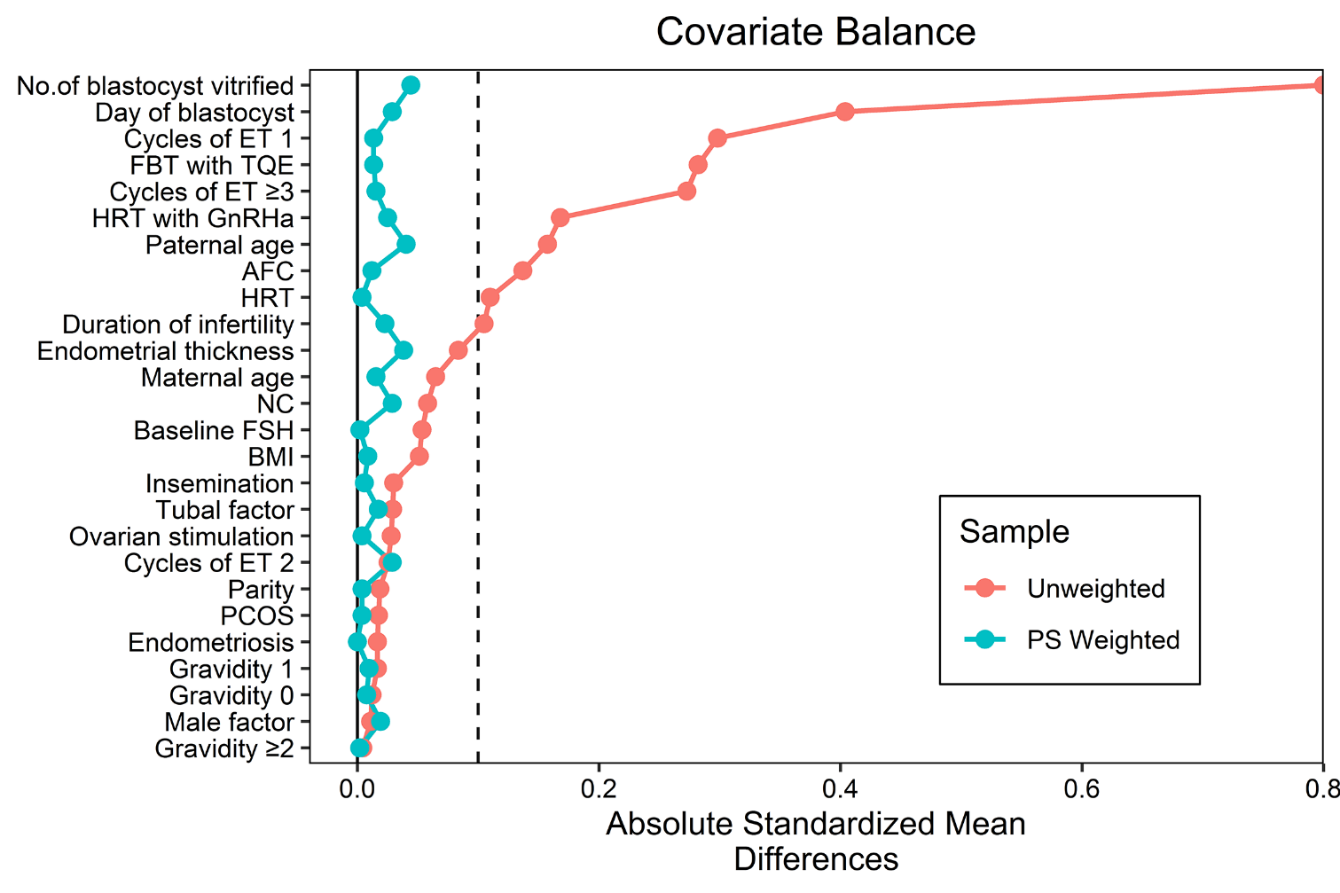


Figure S1 The distributions of the standard differences before and after PS matching were plotted. Standard difference*<*0.1 was used as the threshold to indicate a negligible difference in the prevalence of a covariate between exposure groups
